# Selecting behaviour change priorities for trachoma ‘F’ and ‘E’ interventions: A formative research study in Oromia, Ethiopia

**DOI:** 10.1371/journal.pntd.0007784

**Published:** 2019-10-09

**Authors:** Katie Greenland, Sian White, Katina Sommers, Adam Biran, Matthew J. Burton, Virginia Sarah, Wondu Alemayehu

**Affiliations:** 1 Department for Disease Control, London School of Hygiene & Tropical Medicine, London, United Kingdom; 2 International Centre for Eye Health, London School of Hygiene & Tropical Medicine, London, United Kingdom; 3 Partnerships and Advocacy, The Fred Hollows Foundation, London, United Kingdom; 4 Berhan Public Health and Eye Care Consultancy, Addis Ababa, Ethiopia; 5 Technical Advisor, The Fred Hollows Foundation, Addis Ababa, Ethiopia; RTI International, UNITED REPUBLIC OF TANZANIA

## Abstract

**Background:**

Trachoma is the leading infectious cause of blindness. However, little is known about the behavioural and environmental determinants of transmission of the causative organism, *Chlamydia trachomatis*. We conducted formative research in a trachoma hyper-endemic area of Ethiopia to explore the behaviours which are likely to contribute to trachoma transmission and map their determinants.

**Methodology/Principal findings:**

Data on water use, hygiene, defecation, and sleeping arrangements were collected from five communities during the dry and rainy seasons in 2016. Data collection involved direct observation in households (n = 20), interviews with caregivers (n = 20) and focus group discussions (n = 11). Although several behaviours that likely contribute to trachoma transmission were identified, no single behaviour stood out as the dominant contributor. Hygiene practices reflected high levels of poverty and water scarcity. Face washing and soap use varied within and between households, and were associated with other factors such as school attendance. Children’s faces were rarely wiped to remove nasal or ocular discharge, which was not perceived to be socially undesirable. Bathing and laundry were performed infrequently due to the amount of time and water required. Open defecation was a normative practice, particularly for young children. Latrines, when present, were poorly constructed, maintained and used. Young children and parents slept closely together and shared bedding that was infrequently washed.

**Conclusions/Significance:**

Existing norms and enabling factors in this context favour the development of interventions to improve facial cleanliness as more feasible than those that reduce unsafe faeces disposal. Interventions to increase the frequency of bathing and laundry may also be infeasible unless water availability within the home is improved.

## Author summary

Trachoma is the leading infectious cause of blindness globally. It is caused by *Chlamydia trachomatis*, which is believed to spread eye to eye through direct transmission on skin (e.g. after touching ocular and nasal secretions), indirect transmission on fomites (e.g. towels and bedsheets) and via eye-seeking flies (which breed in faeces). Interventions aiming to interrupt transmission of *C*. *trachomatis* can therefore be designed to promote behaviours ranging from face washing and handwashing to improving sanitation, waste disposal, laundry practices and sleeping practices. Changing behaviour is challenging and interventions are often ineffective if they fail to change important determinants of behaviour. We conducted formative research to understand trachoma-related behaviour and the determinants of these behaviours in order to inform future intervention design. We found that the hygiene practices we observed reflected high levels of poverty and water scarcity. In this context we found that it would be challenging to shift sub-optimal hygiene practices related to latrine use, bathing and laundry without a substantial and large-scale investment in water and sanitation infrastructure. Given this, it may be more feasible for the Government and supporting organisations to design trachoma prevention interventions which target facial cleanliness and hand hygiene.

## Introduction

Trachoma is the most common infectious cause of blindness worldwide. It is caused by the bacterium *Chlamydia trachomatis* but the means by which it is transmitted from eye to eye is not well understood. A number of routes are hypothesised, including: direct transmission on skin (e.g. after touching ocular and nasal secretions), indirect transmission on fomites (e.g. towels and bedsheets) [1] and via eye-seeking flies [2]. As trachoma tends to cluster within villages and households, transmission is also thought to be associated with close, prolonged contact [3, 4]. Cases of active trachoma (trachomatous inflammation, follicular (TF) and/or intense (TI) [5]) are a major source of infection, with the highest bacterial loads usually found in pre-school age children [3, 6].

The World Health Organization’s (WHO) Alliance for the Global Elimination of Trachoma by 2020 (GET2020) aims to eliminate this disease as a public health problem through implementation of the SAFE Strategy: **S**urgery for trichiasis (in-turned eyelid); **A**ntibiotics (azithromycin) to treat active infection; **F**acial cleanliness and **E**nvironmental improvement [7]. In practice, trachoma control programmes have largely focused on the more medical ‘S’ and ‘A’ interventions. This is because these two components are comprised of clearly defined interventions, which are relatively easy to implement and which are supported by a strong evidence-base [8–12]. In comparison, ‘F’ and ‘E’ are outcomes, for which the package of interventions is poorly-defined.

**Fig 1:**
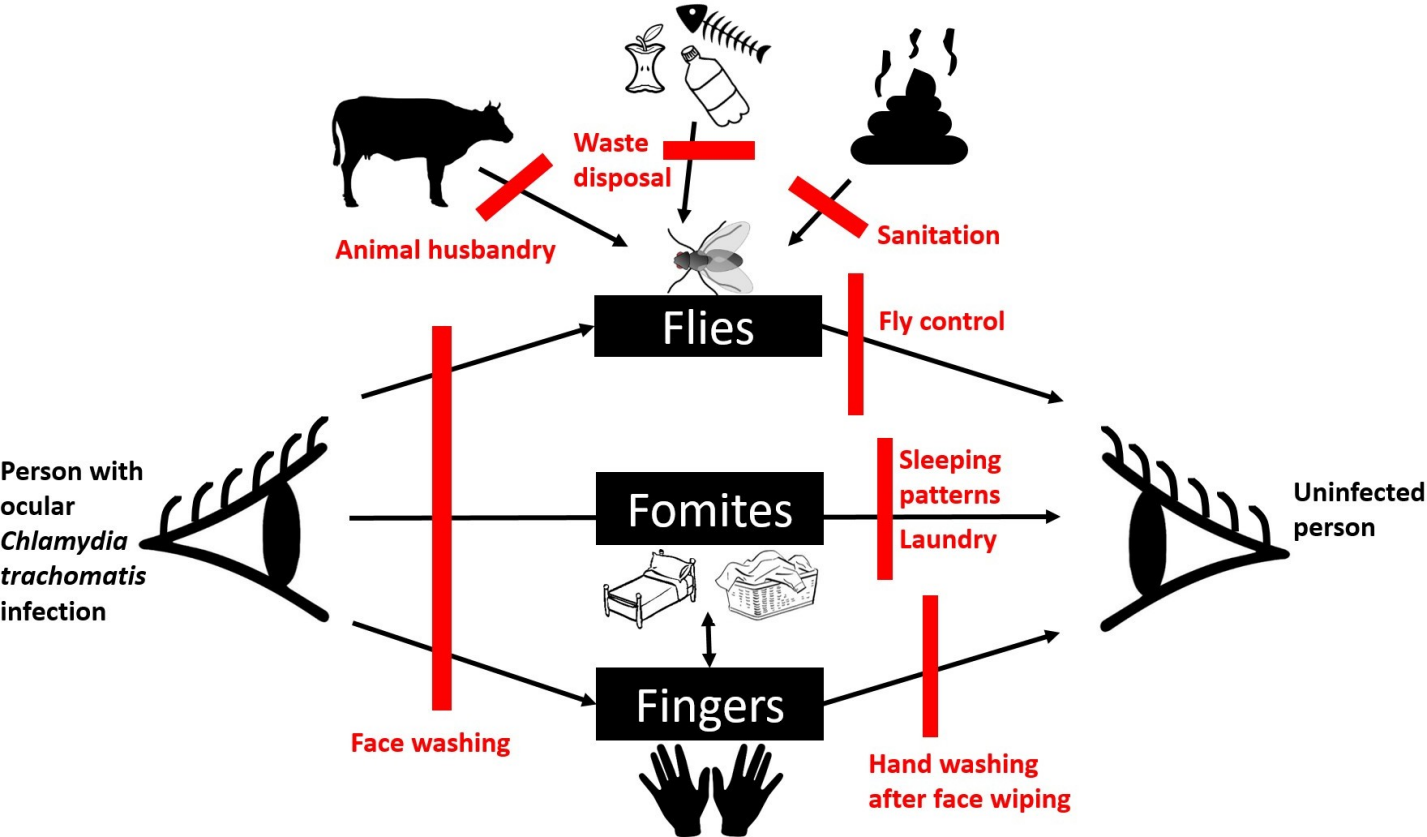
Plausible routes of C. trachomatis transmission (black) and potential “F & E” interventions (red).

The predominant goal of ‘F’ and ‘E’ interventions is to change behaviours associated with an increased risk of *C*. *trachomatis* transmission. In contrast to the other parts of the SAFE strategy, there is a paucity of evidence substantiating the link between ‘F’ and ‘E’ interventions and reduced trachoma transmission. As trachoma could plausibly spread along a variety of routes there is a diverse array of candidate ‘F’ and ‘E’ interventions. These range from the promotion of behaviours like face washing and handwashing to improving sanitation, waste disposal, laundry practices and sleeping practices. Interventions may also address gaps in water and sanitation infrastructure or initiate fly control measures. The heterogeneity of interventions across different trachoma programmes and studies makes it difficult to synthesise evidence in the same way that is applied to medical interventions [13]. This is a common problem in environmental health research. There is a pressing need to build evidence in this area, as antibiotic distribution in endemic regions has not reliably led to substantial reductions in prevalence of active trachoma [1, 14, 15]. Fig 1 portrays an adapted F-Diagram for trachoma transmission based on the work of Wagner and Lanoix [16]. Given the relative importance of these different transmission routes is unknown, it is currently difficult to determine where to focus ‘F’ and ‘E’ intervention efforts. ‘F’ and ‘E’ interventions are also challenging to deliver with high fidelity and coverage and their inconsistent implementation has impeded progress towards trachoma elimination [17]. Furthermore, ‘F’ interventions typically seek to increase knowledge about trachoma rather than addressing more important drivers of behaviour [17].

Formative research provides a mechanism for understanding the determinants of trachoma-related behaviour in a way that can inform future intervention design. The approach has methodological similarities to rapid ethnographies [18, 19] or development research [20]. It uses iterative processes to combine qualitative and quantitative methods in order to generate behavioural insights that are used to inform intervention design. Formative research is relatively rapid and more superficial than ethnographic attempts to understand behaviour [21]. However, these limitations do not invalidate its worth because, as Leviton, et al. (1999) explain, the aim of formative research “is not to answer questions about intervention definitively but, rather, to decrease uncertainty about what the intervention should be” [21]. These studies often go unpublished and there remains a lack of clarity around how formative research objectives are formulated; how methods are identified and used; and how data are used to inform programmatic decisions [22, 23].

This paper describes formative research conducted during two seasons (dry and rainy) to explore household risk factors for trachoma. A review of the literature on trachoma was used to inform our identification of ‘behaviours of interest’. These were: 1) water availability, storage and use; 2) face washing; 3) face wiping and handwashing; 4) sleeping patterns, 5) laundry practices; and 6) defecation practices (including disposal of child and animal faeces) and fly control. The study uses theory-based formative research to select and prioritise feasible behaviour change targets and potential intervention options.

## Methods

### Research framework

The formative research was informed by Behaviour Centred Design (BCD) [24]. BCD draws on evolutionary and environmental psychology in order to define and explore critical domains of behaviour including cognitive processes and the way individuals interact with their environments. The BCD checklist of behavioural determinants was initially used to generate research questions about the behaviours of interest (Table 1). Methods were then developed to investigate these research questions. BCD has a set of recommended formative research methods [25], but the approach encourages users to create their own methods if the existing tools are unable to sufficiently answer the behavioural questions of interest [26]. As we were unable to identify suitable tools to explore some behavioural determinants in other trachoma formative research studies and guidelines [27, 28], we developed a set of additional participatory activities that were conducted as part of interviews or focus group discussions (FGDs). The methods employed in this research and reported on in this paper are described in Table 2 and under ‘Procedures’. The BCD determinants under study are also presented in Table 2.

**Table 1:** Research questions derived from the BCD determinant checklist [29] and related to the behaviours of interest.

	Key research questions related to the behaviours of interest
**Psycho-social** *(Including knowledge*, *beliefs*, *capabilities planning*, *identity*, *routines*, *perceived norms*, *motivations*, *habits and discounts/trade-offs)*	• What do people know about trachoma? • Do the audience understand the need for the behaviours of interest and when and how it should be done? • Do people feel like they have the ability to practice the behaviours of interest? • What motivates or demotivates the community to practice the behaviours of interest? • Are the behaviours of interest rewarding? • How socially important is it to be clean in order to fit in within the community or at school? • What cues the behaviours of interest? • Who performs the behaviours of interest? • How is the performance of the behaviours linked to their role or identity? • 10. What activities happen before and after the behaviours of interest? • 11. Where do the behaviours of interest fit within daily routines? • 12. Do people normally practice the behaviours of interest? • 13. What behaviour is socially expected or socially desirable? • 14. What compromises or trade-offs do people make in relation to the behaviours of interest • 15. How are soap and water prioritised? • 16. How much water do people perceive to be necessary for the behaviours of interest? • 17. What circumstances distract people from being able to practice the behaviours of interest?
**Environment** *(including infrastructure*, *objects*, *the physical setting where behaviours take place*, *contamination in the environment and the social environment)*	• Do practices vary by season?19. How does the geography of the region affect the behaviours of interest? • Where do the behaviours of interest take place? • What objects are used to perform the behaviours of interest? • What attitudes are held towards these objects? • How much water is collected on average per family? • What other infrastructure is necessary to perform the behaviours of interest? • What sources of contamination are there in the environment? • Are people worried about trachoma? • Are people bothered by the presence of flies? • Is there social pressure to practice the behaviours of interest? • How does the social environment (relationships, networks and organisations) affect the behaviours of interest?

**Table 2:** Methods informed by the Research Questions and BCD formative research guide [25].

Method	Description	Psycho-social (PS) and Environmental (E) Determinants explored	Sample
Extended unstructured observation	Observation: Direct observation of household activities of all household members from dawn for up to 8 hours. Accompanied family members to the water point to collect water.	Physical environment (E); Infrastructure (E); Objects (E); Environmental contamination (E); Habits (PS)	20 observations, 10 dry season and 10 wet season
	Mapping: Maps of each household were drawn and researchers marked the presence of faeces and where behaviours of interest occurred (e.g. face washing and handwashing).	Physical environment (E); environmental contamination (E)	
Post-observation interview	Routine Scripting: The female caregiver was asked to list the activities of her daily routine, with a focus on activities performed outside of the observation period. Women were also asked about the things that cause routines to vary.	Routines (PS)	13 female caregivers
	Mapping: The sleeping arrangements for all household members were mapped.	Physical environment (E)	
	Soap inventory: Participants produced soap available in the house and discussed its uses and attributes.	Objects (E); Discounts (PS)	
	Water prioritisation: Participants used cups to explain how water is allocated to different household tasks and how water availability alters water use.	Infrastructure (E); Discounts (PS)	
	Socio-demographic questionnaire: Socio-demographic information was collected.	Characteristics	
Focus group discussion	Mapping: A large map of a ‘typical’ house was used to determine where in the household and its surroundings different family members were likely to sleep and to defecate.	Physical environment (E); Environmental contamination (E); Norms (PS)	4 FGDs with mothers with a child under 3
	Identity drawing: Participants described the ‘ideal’ woman in terms of their physical appearance, personality traits, their role within the community and their hygiene behaviours	Norms (PS); Identity / role (PS)	
	Feelings: Participants described the times of day when they feel happy/content/confident in themselves and the times of day when they feel uncomfortable/unhappy/ or lack confidence in themselves. They then described how they feel after performing each behaviour of interest.	Motives (PS); Routines / habits (PS)	2 FGDs with older women
	Illness severity ranking: Participants ranked cards depicting a range of illnesses and impairments in order of severity and discussed their consequences	Knowledge / beliefs (PS); Discounts (PS); environmental contamination (PS)	
	Disgust scaling: Participants ranked a range of items, including things related to the behaviours of interest such as animal faeces, human faeces, dirty hands, eye discharge, and nasal discharge in order of disgust.	Motives (PS); habits (PS)	
	Knowledge of trachoma transmission: Open discussion about trachoma and its causes, prevention and treatment.	Knowledge (PS); Capabilities (PS)	All 11 FGDs
	Role shifting: Documentation of household roles and potential for role shifts.	Roles (PS); Capabilities (PS)	2 FGDs with male heads of household

### Study setting

This research was carried out in the Oromia Region of Ethiopia in January (dry season) and July (rainy season) in 2016. Data were collected from five communities in Wore Jarso district, an area with high prevalence of active trachoma (TF = 30%-50% in children 1–9 years) [30] bordering the Amhara Region. Water scarcity is a major challenge in rural Ethiopia: over half of the population travel more than 30 minutes to collect water [31]. Only 4% have access to a ‘safely managed’ source of drinking water–defined as an improved water source located on premises, available when needed and free from faecal and priority chemical contamination; open defecation is common (27% of the rural population) [32]. Study communities were selected purposively to reflect variations in sanitation and water access. Data were collected in two seasons because it was anticipated that the behaviours of interest were likely to vary during these different times of year due to differing water availability and daily routines.

### Procedures

#### Extended unstructured observations and interviews

Two non-neighbouring households in each community were identified through a random walk. Eligible households were those with at least one child aged 1–9 years. Households were recruited around dawn on the day of observation to prevent households from cleaning or otherwise preparing for the study. Where possible, the same households were observed during both seasons. To minimise reactivity, participants were not told which specific behaviours we were interested in, rather they were informed that we were interested in learning about their daily routines. Observation took place for up to 8 hours, from around 7am until between noon and 3pm, depending on participants’ farming activities. Two female researchers were stationed in each household, moving within the house and compound as needed to monitor behaviour. Each pair comprised a foreign researcher and a local research assistant. At the end of each observation, observers compared notes. If family members were in multiple locations, emphasis was placed on observing locations where water was present so that performance of hygiene behaviours would not be missed. Whenever possible, one researcher accompanied family members to collect water. Photos were used to capture behaviour in context, and video was taken to help understand sequences of behaviour. Maps of each household were drawn and the presence of faeces and location of occurrence of the behaviours of interest (e.g. face washing, defecation) were marked.

At the end of each observation period, the female primary caregiver was interviewed and the socio-demographic questionnaire was completed. Interviews followed a topic guide and included props to explore daily routines, sleeping arrangements, soap availability and attributes, and water prioritisation and use. Participants in households observed during both seasons were interviewed twice.

#### Focus group discussions (FGDs)

FGDs were held across the four communities over the two seasons (Table 2). FGDs were separately conducted with mothers of children under three years-of-age, male household heads, grandmothers and 15 and 16 year-old sisters of pre-school children (rainy season only). These population groups were selected because they all play a role in caring for young children (who have the highest rates of infection). The FGDs were held with similar individuals (age and gender) and the size was kept small (5–8 individuals per group) to encourage active participation. Participants were selected by health extension workers who were instructed to invite people with different levels of wealth and education and from different areas of the communities. Multiple members of the same family were not included and there was no overlap between the populations included in the household observation and FGDs. FGDs were designed to build consensus around themes that had arisen from observation and individual interviews and to explore some additional behavioural determinants. Tools were used to encourage participants to make generalisations about the perceptions and practices of their community.

#### Data handling and analysis

All FGDs and interviews were voice-recorded and transcribed into English. Observation notes were typed up. A two-step process was used for the analysis in line with the BCD approach. During data collection, the team adopted a process of ‘sequential recycling’. This is a process drawn from social marketing whereby the investigators come together at the end of each day to discuss the findings [26]. Each team member contributes anecdotes or patterns they noticed during data collection. These items are then sorted into BCD checklist categories, and discussed. When there is agreement, the item becomes a finding. Where there is disagreement or if knowledge gaps remain, then questions are developed for further exploration. This process also allowed us to identify when we had reached a point of saturation suitable to meet the study objectives. At the end of data collection a deeper thematic analysis was conducted using the BCD determinant checklist [29] as a deductive ‘top down’ coding system in relation to each of the behaviours of interest [33] (coding tree included in supplementary materials, S1 Fig). The coding was performed by two authors (KG and KS) and reviewed by a third author (SW). Where disagreements occurred the findings were discussed between all authors in order to develop a coherent thematic summary.

#### Ethics and consent

The research protocol was approved in Ethiopia by the Oromia Regional Health Bureau’s Research Ethics Review and by the London School of Hygiene & Tropical Medicine Ethics Committees. Informed written consent was obtained for each household member 18 years or over. Parents consented on behalf of children under 18 years, but those aged 7 to 17 years also provided assent.

## Results

### Characteristics of study population

A total of 13 households participated in the observation component of the study and a further 41 women, 14 men and 6 young women (15 and 16 years of age) participated in FGDs. The first round of formative research took place over two weeks in January 2016 (dry season) and coincided with *Timkat* festivities. The repeat study took place over four weeks in July 2016 and overlapped with school holidays, which last from June-September.

Three of the 10 recruited observation households could not be revisited in the rainy season due to inaccessibility (n = 1), family breakdown (n = 1) and relocation (n = 1) and were replaced by neighbouring households from the same communities. All respondents were married, had low levels of literacy and were Christian (mostly Coptic Orthodox). Compounds were clearly demarcated with fences. All households kept animals and had land for farming. Communities differed with respect to latrine access and water access, as well as electricity (lighting) and radio ownership (Table 3). FGD participants came from the same communities as the observation households and likely had similar living conditions and water and sanitation access.

**Table 3:** Profile of households observed during the study.

	Community A				Community B				Community C				Community D				Community E			
ID	DS:1	RS:1	DS:2	RS:2	DS:3*	RS:3*	DS:4	RS:4	DS:5*	RS:5*	DS:6*	RS:6*	DS:7	RS:7	DS:8	RS:8	DS:9	RS:9	DS:10	RS:10
HH size	9	7	5	5	5	6	8	5	8	7	8	11	7	5	7	10	9	9	6	6
No. children <5yrs	0	0	1	1	0	1	1	1	2	0	1	2	0	0	2	1	2	1	1	1
No. children 5-17yrs	3	2	2	2	3	3	4	2	4	2	5	6	3	3	4	6	4	5	2	2
Estimated vol. water/person/day	19L	9L	15L	16L	15L	7L	19L	8L	8L	11L	9L	9L	17L	8L	6L	4L	8L	7L	13L	8L
Latrine	No	No	No	No	No	No	Yes	Yes	Yes	Yes	Yes	Yes	Yes	Yes	Yes	Yes	No	No	No	No
Human faeces in compound	Yes	Yes	Yes	Yes	Yes	No	No	No	No	No	No	No	Yes	No	Yes	No	No	Yes	Yes	No
Soap available in home	Yes	Yes	No	No	Yes	No	Yes	Yes	Yes	Yes	Yes	Yes	Yes	Yes	No	Yes	Yes	Yes	Yes	Yes
Electricity (light)	Yes	Yes	Yes	Yes	No	No	No	Yes	Yes	No	Yes	No	Yes	Yes	No	No	No	No	No	No
Radio	No	No	Yes	Yes	No	No	No	No	Yes	No	No	Yes	Yes	Yes	No	No	No	No	Yes	Yes

* Different households were included in the rainy and dry season studies

Eleven focus groups were held across the five communities with groups of mothers with children under three (n = 6), male heads of household (n = 2), older women (n = 2) and teenage sisters of pre-school children (rainy season only).

### Water availability, storage and use

Water was primarily used directly from the water containers it was collected in. Little water remained in households overnight, with the exception of any rainwater in the rainy season. Consequently, water collection was one of the first activities of the day. Households in the same community often chose different water points due to cost, perceptions of cleanliness of water, and convenience. All but one household collected water from public water points, either a borehole or a protected dug well, which were locked in the afternoon and subject to daily collection limits. Public water points were rarely more than 15 minutes walking one way; however, in the dry season, wait times of up to one hour were experienced. Water was also collected from unprotected springs and the river during the dry season. Eight out of the 10 households collected rainwater for household use during the rainy season. Daily household water availability varied from 6 to 19 litres of water per household member during the dry season. Rainwater harvesting in the rainy season partially replaced rather than supplemented water collection, resulting in very similar water availability per head (4 to 16 litres). As households also had livestock, the amount of water available per capita should be considered to be somewhat less than this.

Water was used sparingly for cooking, washing dishes, preparing coffee and providing drinking water for animals in both seasons. Other activities took place predominantly or exclusively in the dry season: washing clothes, bathing young children, relaying the floor with cow dung and watering banana plants. The presence of guests and occurrence of celebrations and special events tended to increase water collection and use.

### Face washing

#### Current practices

**Fig 2:**
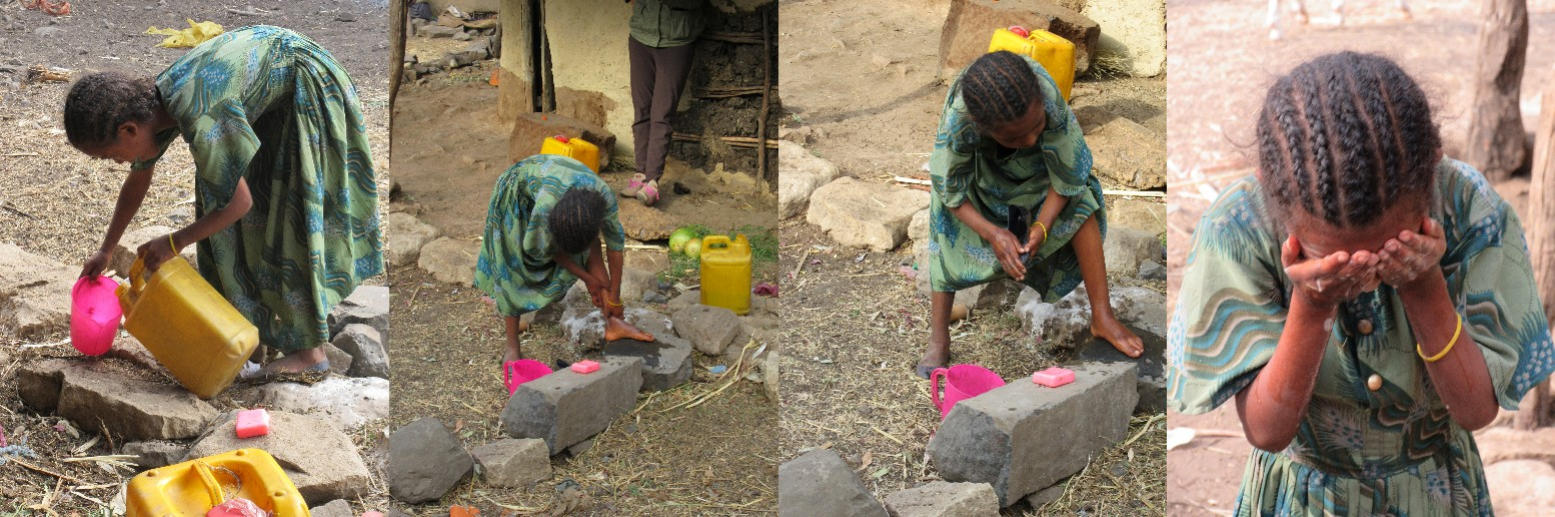
Girl washes with soap before school during a typical morning washing routine involving the feet, legs, hands, arms and face.

Body washing took place outside shortly after waking using a small bucket or pail of water filled from a larger water container. Faces were washed at this time, along with feet, legs, hands and arms in a similar manner to that depicted in Fig 2. Faces were not dried after washing. Overall, 33 of 63 (52%) individuals in the dry season and 21 of 67 (31%) individuals in the rainy season were observed washing their faces. Around two thirds of individuals used soap during face washing (64% dry season, 62% rainy season), but soap use was inconsistent within as well as between families. There were no dedicated places for face washing, but faces were typically washed outside. Face washing was only observed on one other occasion (after a child had eaten oily food), but is reportedly carried out again later in the day (after work or school, or before bed).

**Fig 3:**
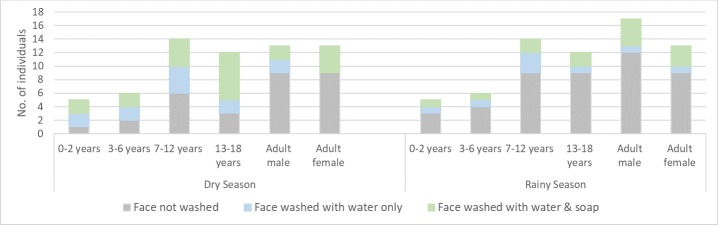
Observed face washing behaviour amongst household members of different ages in the dry and rainy season.

Face washing practices varied by age and season (Fig 3). During the dry season the majority of school children washed their faces, but there was a marked decline in face washing amongst school-going children during the rainy season. Adults washed faces less frequently in both seasons, but tended to use soap when they did wash. Pre-school children as young as three years-of-age were observed washing independently and unprompted, sometimes with soap. Only the faces of the youngest children were washed directly by their mother, on five occasions (four in the dry season) as part of a full bath which used minimal water (<1litre). No bathing was observed among older family members, corresponding with reports that school children bathe weekly and adult women may bathe as seldom as every two months.

#### Environmental and psycho-social determinants

In contrast with observed practices (Fig 3), mothers state that children’s faces are washed as a priority:

“*He doesn’t go without eating breakfast*, *so that means he can’t go without [face] washing”*. *(Mother*, *Dry Season Interview*)

Face washing was perceived to be rewarding, supposedly giving children a positive start to the day and promoting sleep, responses that align with observed face washing in the morning and reported face washing before bed.

*“It feels uncomfortable if their face is not washed; it refreshes them and gives them a brighter day and mind and they can also sleep easily*.*” (Mother*, *Dry Season Focus Group*)

Although participants associated unclean faces with flies and disease transmission, pre-school children’s dirty faces were never spontaneously washed, so it is likely that face washing is driven more by habit and less by presence of discharge. This may also explain why all but the youngest pre-school children (who received the prioritised attention of their mother), did not always automatically wash their faces in the morning. The observed independence of body washing by age three differs from the collective opinion of mothers that children need assistance with their personal hygiene until they are around seven years old.

School attendance appears to be a strong driver of face washing (with soap). It is a requirement to have a clean face for school, and children are reportedly disciplined and even sent home from school if their faces are dirty. It is unclear whether dirty children are judged negatively by others in the community as women were divided about the extent to which people would gossip about them or their ‘dirty’ children.

*“People gossip about you even if they don’t tell you. They may say: ‘Why did she leave her child with a dirty face?’” (Mother*, *Dry Season Focus Group)*

*“Everyone thinks it is up to the mother*, *they don’t interfere in someone else’s business*.*” (Mother*, *Dry Season Interview*)

The observed reduction in face washing among school-age children during the rainy season may be due to a perceived reduction of this school-related social pressure during the school holidays. However, the rainy season is also a busy time agriculturally and it is not considered necessary or feasible to remain clean when time is predominantly spent working in the fields and you are not seen socially.

*“During school [students] …want to be seen as attractive*, *but now [during the rainy season] we all go directly to the fields each day instead*, *so the frequency of washing has declined*.*” (Mother*, *Rainy Season Focus Group*)

*“We go home when it gets dark*, *we don’t even think of what we eat during those seasons*, *we don’t even wash our legs*.*” (Mother*, *Rainy Season Focus Group*)

Water collection reflects planned activities for the day, and water is used conservatively: mothers were seen to chastise children for wasting water whilst washing. Soap use requires additional water use, so water scarcity may limit face washing with soap. As soap is kept inside, but washing takes place outside, lack of soap at the location of use may prove more limiting than the overall lack of soap. In these communities only two of the families had no soap present in the household in each study. Participants only considered soap to be necessary for face washing when a face is really dirty, which was not a cited key reason for face washing. This may further explain why soap is inconsistently used.

### Face wiping and handwashing

#### Current practices

Many pre-school children in the study households had nasal or ocular discharge on their faces during the observation period; flies on faces were also abundant. Discharge was removed from children’s faces on only 14 occasions across both data collection periods. Children only occasionally wiped their own faces (on their sleeves or using their fingers). Faces were instead wiped by female caregivers using hands which were then wiped on clothing or a nearby surface. We observed a child’s garment being used for wiping on one occasion only, but the use of clothing is reportedly more common when mothers are working in the fields and have dirty hands.

Handwashing is important in this context because it is plausible that *Chlamydia trachomatis* is transferred from faces to hands during face wiping, facilitating transfer to another face. Handwashing was seldom witnessed other than as part of the morning washing routine. Some handwashing prior to and after eating was observed, but hands were never observed to be washed after wiping facial discharge, after handling child faeces, or after returning from defecation. Soap was not observed to be used for handwashing during the rainy season and was only used on 6 of 37 (16%) occasions during the dry season—all but once after eating.

#### Environmental and psycho-social determinants

The presence of flies and discharge on children’s faces rarely cued face wiping. However, requesting to take a photo prompted discharge to be removed from children’s faces. This suggests that while discharge is tolerated, it is still considered socially undesirable. Participants felt that their environment constrains them from acting any differently:

*“We usually wipe [a child’s face] with our hands and put [the discharge] on the ground*. *We don’t have anything else to use because we are in a rural area*, *but it is not enough to be clean*.*” (Mother*, *Dry Season Focus Group*)

Handwashing after face wiping is not the social norm. Soap use patterns also reflected opinions that soap is only necessary when hands are sticky or visibly dirty. The lack of handwashing after face wiping suggests that discharge is not viewed to be sufficiently dirty or disgusting to cue handwashing.

### Sleeping patterns

#### Current practices

Study participants slept communally on raised beds, straw mattresses, or goat skins. Generally, young children slept together with their parents or grandparents, whilst older children slept apart. Blankets were generally shared and although pillows were not commonly used, old garments or grain bags were sometimes used by older family members. Skins, mats and blankets used for sleeping were tidied away each morning or were put outside to air. Overnight guests are provided with clean bedding that is reserved for special occasions.

#### Environmental and psycho-social determinants

Young children sleep together with their mother because they can cry at night, because it is often cold and because of the limited number of blankets available. The cost of purchasing a new blanket is seen to be prohibitive (250–300 Ethiopian Birr, around US$10). Guests are given preferential treatment:

*“We don’t give them [guests] the blankets we always sleep on and use because we respect them*.*” (Grandmother*, *Dry Season Focus Group*)

### Laundry practices

#### Current practices

Clothing was washed in three of the ten households observed in the dry season (twice involving only an infant’s clothing, once a full load of laundry) and not at all during the rainy season. Observed behaviour contrasts with reports that the clothing of children under two is prioritised for washing and this occurs at home on a daily basis. School clothes are reportedly washed weekly, but adult clothing may be washed infrequently (bi-monthly or less), while blankets are rarely washed. Some respondents reported doing laundry at their home while others did it at nearby rivers. Rivers or other water bodies tend to be used to wash large items such as adult clothes and bedding which use more water.

#### Environmental and psycho-social determinants

Participants thought that bedding should be washed or aired when it becomes “itchy”, but were unaware of any connection between dirty clothing or bedding and disease transmission. Laundry is considered taxing in terms of water volume required (70 litres was observed to be used to wash a load of both adult and child clothing), soap used and time. Effort is made to clean clothes reserved for special occasions (*Timkat* celebrations in the dry season) as they are worn in public. Given the limited availability of water at home and effort required to wash at the water source, laundry of clothing of infants and school children are prioritised because the former become soiled frequently and the latter need to look clean and smart for school. Laundry is deprioritised in the rainy season, when clothing takes longer to dry, water sources are muddy and life is too busy:

*“We don’t usually wash our clothes regularly during the farming season [rainy season]*. *We work a lot in the field and go home when it gets dark*. *We don’t want to lose time by washing clothes; we don’t even have time for sitting*.*” (Mother*, *Dry Season Focus Group*)

### Defecation practices and the environment

#### Current practices

If human faeces are not safely contained in a latrine they may become a breeding site for *M*. *sorbens* flies [34]. Half of the households under observation in each season had a simple pit latrine, but these latrines were poorly constructed, with shallow pits, limited or no superstructure and small holes which made them difficult to use hygienically. Latrines were reported to be washed away in the rainy season, but this was not observed. Although many latrines were visibly in use, the presence of human faeces within the same compounds suggested that latrines were not used exclusively by the whole family. The age at which children were reportedly able to use a latrine varied from seven to 12 years, depending on whether an adult’s assistance was required. Women expressed concern over young children falling into the hole or playing with faeces. The youngest children were permitted to defecate freely within the immediate compound, including adjacent to the living structure and child defecation within the compound was witnessed on six occasions during the study, after which the faeces were left *in situ* until dry. Hours later it was swept up and disposed of with other rubbish in a pile in a specific area of the compound. Adults and older children who open defecated reportedly did so before dawn (behind the house or in a nearby field), or later in the day whilst working in the fields. These faeces were not reportedly covered or moved. Animal faeces may also be used for breeding by *M*. *sorbens* flies [34]. Animal faeces were also scattered throughout all of the compounds studied, as well as within the home or kitchen in houses where animals slept in the family’s living quarters. Cow dung was reused for household purposes, such as relaying the floor.

#### Environmental and psycho-social determinants

Latrine building is encouraged by Health Extension Workers for health reasons, but is principally motivated by the desire to avoid a fine. Poorly constructed latrines with limited superstructure and small holes offered little privacy or enticement. Experiences of latrine use were often negative, with reports of bad smells and not feeling good after using a latrine. As such there was little demand for sanitation and few examples of hygienic public latrines (the schools and health centres visited had well-constructed, but poorly maintained or non-functional facilities). In contrast, open defecation was viewed as acceptable, comfortable, convenient and beneficial for the land:

*“Faeces helps trees to grow if it is thrown in the field and it helps the land to become fertile; It [faeces] does not have a benefit at home [in a latrine]*.*” (Mother*, *Dry Season Focus Group*)

Little effort was made to try to remove animal faeces from compounds, possibly because animal faeces were viewed to be less disgusting than human faeces and because it was an important source of fuel.

The presence of flies was linked to “disease”. However, HIV was listed as a disease that could be spread by flies, along with diseases such as diarrhoea and trachoma. The presence of flies was thought to depend on the weather, but was also linked to fresh milk, cattle, uncovered food, faeces, dead animals, the latrine, and other dirty things in the environment:

*“Flies do not land on a clean thing*, *on a clean face*, *so we have to clean our environment*.*” (Father*, *Rainy Season Focus Group*)

That said, participants did not believe that there was much they could do to control flies.

## Discussion

This formative research provided a rapid assessment of a large number of behaviours that may facilitate trachoma transmission in the study setting. Here we reflect on the study findings and explore relevant behaviour change goals in this context. To aid selection and prioritisation of intervention targets, consideration is given to both the feasibility of altering a given behaviour and the biological plausibility that behaviour change would reduce *C*. *trachomatis* transmission.

Water is scarce in this setting and was observed to be used conservatively. However, participants reported having sufficient water to meet their needs and were able to adjust their water collection and consumption according to their activities in both the dry and rainy seasons. The similar level of household water availability in both seasons was surprising and contrary to expectations and other findings from the region [35]. However, total water availability is not always linked to water use for washing [36]. This suggests that water could be used to perform behaviours promoted by an intervention if an intervention convinces the target population that the desired behaviours should be prioritised and demand little water.

### Face washing and wiping to improve facial cleanliness

Half of household members routinely washed their faces in the morning in the dry season and a third did so in the rainy season when long school holidays and intensive periods of farm work appeared to interrupt morning routines. Two-thirds of those washing faces used soap and almost all households had soap. Soap availability varies from community to community [27, 37, 38], but is not a major barrier to face-washing in the study setting. Whilst the available evidence in support of face washing as an effective means of trachoma control is inconclusive [12], clean faces (defined by their lack of nasal or ocular discharge [39]) and daily face washing are associated with reduced odds of *C*. *trachomatis* infection [11]. As illustrated by Fig 1, ensuring that faces are free of discharge is plausibly the most important means of interrupting trachoma transmission. Face washing is likely to be most effective at removing nasal and ocular discharge if soap is used and if it is practised frequently. As primary school and pre-school children least often washed their faces (with soap), yet experience the greatest burden of active trachoma [40], it is logical to target facial cleanliness interventions towards improving practices among these groups. It would be relevant to encourage family members with existing face washing habits to assist young children who are not young enough to be washed as a priority by their mothers.

Face washing needs to be linked to the daily routine rather than the school routine, as the school holidays appear to have interrupted good habits in this context [41]. It may also be possible to use the daily routine to encourage face washing on additional occasions throughout the day. The creation of face (and hand) washing stations where soap and water can be kept and used should be explored to make face washing easier and more observable. Whilst face washing during bathing is actively promoted by some hygiene interventions (e.g. Super School of 5 [42]), the barriers to frequent bathing identified by this research do not make this a viable way of ensuring faces are washed in this setting.

Face washing should ideally be promoted in conjunction with regular face wiping, as discharge likely builds up on faces in between face washes [43]. As face wiping is rare but typically involves the use of hands, any efforts to improve the frequency of face wiping would also need to target subsequent handwashing, a behaviour that is not currently triggered by face wiping. It is possible that a child’s face is dirty so often (even shortly after wiping away discharge), that face wiping is regarded to be futile by carers in this setting. Given the difficulties associated with changing multiple behaviours [44, 45] and the lack of knowledge about the effectiveness of a face wiping and handwashing intervention, it may be more sensible to focus intervention efforts on improving face washing practices. To overcome this and improve the frequency of face wiping, it may be possible to enhance emotional responses (disgust and associated social sanctions) to the presence of nasal and ocular discharge [46, 47]. A heightened disgust response might also facilitate handwashing with soap after face wiping.

### Laundry to reduce fomite-related transmission

Clothing and bedding were washed infrequently in this context. We did not identify other formative studies that have studied these behaviours in relation to trachoma. In this setting it seems unlikely that people would be able to substantially change their clothes-washing behaviour, let alone the washing of bedding, in the absence of a water supply intervention that would increase water availability per capita. However, it may be possible to build on the existing washing habits for infant clothing to prioritise the washing of clothes more likely to come into contact with nasal and ocular secretions, such as the clothing of pre-school children or scarves.

### Altering sleeping patterns to reduce direct transmission at night

As family members slept closely together and shared blankets, direct transmission of *C*. *trachomatis* may occur at night time. Focus group participants did not consider it possible to alter sleeping patterns (e.g. asking children to sleep “top and tail” or to sleep separately). Interventions that could remove *C*. *trachomatis* from the sleeping environment may be worth exploring, for example, the promotion of individual pillows that can be washed or exposed to the sun each day. However, pillows are not currently used by children, so it may be more feasible to ensure that faces are clean before going to bed at night than to introduce another new behaviour.

### Sanitation and fly control to reduce the abundance of *Musca sorbens* flies

The ‘E’ component of the SAFE strategy in practice mainly involves increasing household access to water and sanitation services, as well as, in some cases, increasing community coverage of these services. The former because water availability at the household level is strongly associated with the ability to practice hygiene behaviours [48], the latter because of the anticipated role of *M*. *sorbens* flies in trachoma transmission [2, 49] and their preference for human faeces for development of immature stages [34]. While there is also some evidence that fly populations can be reduced through insecticide spraying and sanitation [50, 51], these effects have not been seen in all settings [9]. Given the observed high prevalence of open defecation, the closeness of contact between animals and humans, the abundance of both animal and human faeces in homes and compounds and the abundance of flies, it is likely that a combination of the interventions shown in Fig 1 (sanitation, animal husbandry, waste disposal and direct fly control) would be required to reduce the presence of *M*. *sorbens* flies in this setting. Since animals were commonly kept within the home to protect them from theft and the elements, it may be difficult to bring about significant changes to animal husbandry practices that would substantially alter the presence of animal faeces and thus flies in compounds. Improving latrine access, quality and use may be a more viable option but, in order to be successful, programmes must address current negative attitudes towards latrines, a known barrier to latrine uptake and use [52, 53]. Direct fly population control interventions such as use of repellents and insecticides were not explored in detail in this study, but participants indicated that they would be receptive to these types of interventions. The impact of repellents on fly control and trachoma has not been studied and the available evidence for the impact of insecticides on these outcomes is inconclusive [50, 54].

### Reflections on the formative research process

This research serves as an example of how to conduct theory-based formative research on a topic where limited prior evidence existed. One of the strengths of using the BCD framework to guide the formative research process was that the determinant checklist forced us to explore aspects of behaviour that we may have otherwise overlooked. Secondly, the approach emphasises the primacy of observational data which is then supplemented with methods to understand local rationalisations for behaviour. Our findings show that this combination provided a much more complete picture of behaviour than we would have obtained without the observational data. The triangulation of observational data with self-reported descriptions and reflections on behaviour highlighted inconsistencies in relation to several behaviours of interest. We feel that these inconsistencies are indicative of the daily tensions that play out at individual and community levels as people make trade-offs between their perceptions of socially desirable behaviour and their inability to consistently practice such behaviours in a setting with scarce resources and multiple competing priorities. Lastly, the BCD approach conceptualises FGDs and interviews as merely structures through which to undertake participatory activities. The use of drawing, mapping, image prompts and props throughout this research made it more engaging for both the research participants and the research team and allowed the tools to work effectively among a low literacy population. As with any rapid research process there was a tension between what was feasible for us to explore in a short timeframe and the appeal of a deeper, broader understanding of the socio-cultural world in which these behaviours take place. In this case, these tensions were reconciled by the knowledge that this work was the starting point to inform longer term mixed-method research on this topic.

Our approach was limited by the fact that observations were only done during part of the day and among a small sample. For security reasons it was not possible to observe evening washing routines, so our understanding of behaviour at this time is based on self-report and needs to be verified. Seasonal changes in daily routines would also benefit from further verification as the *Timkat* holiday (dry season) and school holidays (rainy season) may have affected behaviour. We have recently completed a study in a cohort of households followed over two days and a night during three seasons with a view to bridging some of these gaps. Behaviour may have been influenced by the presence of foreign researchers, but the lack of occurrence of ‘desirable’ behaviours suggests that this was not a major source of bias. Repeating the study in the same households may have also led to reactivity during the second study (rainy season). However, six months had passed, the research was conducted by different individuals, and face washing was less frequently observed at this time, which collectively suggest that reactivity was not a particular concern in this study. Water availability per capita per day was calculated using reported information on water collection coupled with observed water collection. These estimates reportedly vary day to day depending on the activities being conducted. There is also likely to be some inaccuracy in estimates of rainwater availability due to discrepancies in reporting and the diversity of containers used to collect rainwater. Rapid formative research still requires detailed analysis in order to maximise the findings. The quality of transcripts produced from the FGDs and interviews may have jeopardised the quality and richness of our learning as our analysis ended up more heavily based on observational field notes than transcripts. If replicating research like this, we would recommend using multiple transcribers so that the translation team can cross-check each other’s work and monitor quality. Stationing two researchers in each household for the observations minimised the likelihood that behaviours of interest went undocumented. This research explored a large number of behaviours and determinants. We were unable to fully describe the relationships between all determinants and the behaviours of interest in this manuscript.

### Concluding remarks

This study suggests that it is possible to use participatory formative research based on behavioural theory to help define intervention targets and shape the content of sensible, theory-based interventions that could plausibly change behaviour and thereby improve the effectiveness of “F & E” strategies for trachoma control. Specifically, this research indicates that in this region, without a substantial and large-scale investment in water and sanitation, the most viable behaviours to target to reduce trachoma transmission are face washing and handwashing with soap. We are conducting ongoing microbiological and behavioural research into the transmission dynamics of trachoma in this region. This may improve our ability to design interventions that are more likely to interrupt transmission.

## Supporting information

S1 Fig Coding tree used in the thematic analysis. (TIF)
