# Supplementary figures and images for: Selecting behaviour change priorities for trachoma ‘F’ and ‘E’ interventions: A formative research study in Oromia, Ethiopia
Source: PLoS Negl Trop Dis. 2019 Oct 9;13(10):e0007784. doi: 10.1371/journal.pntd.0007784 (PMC6785218; doi:10.1371/journal.pntd.0007784)

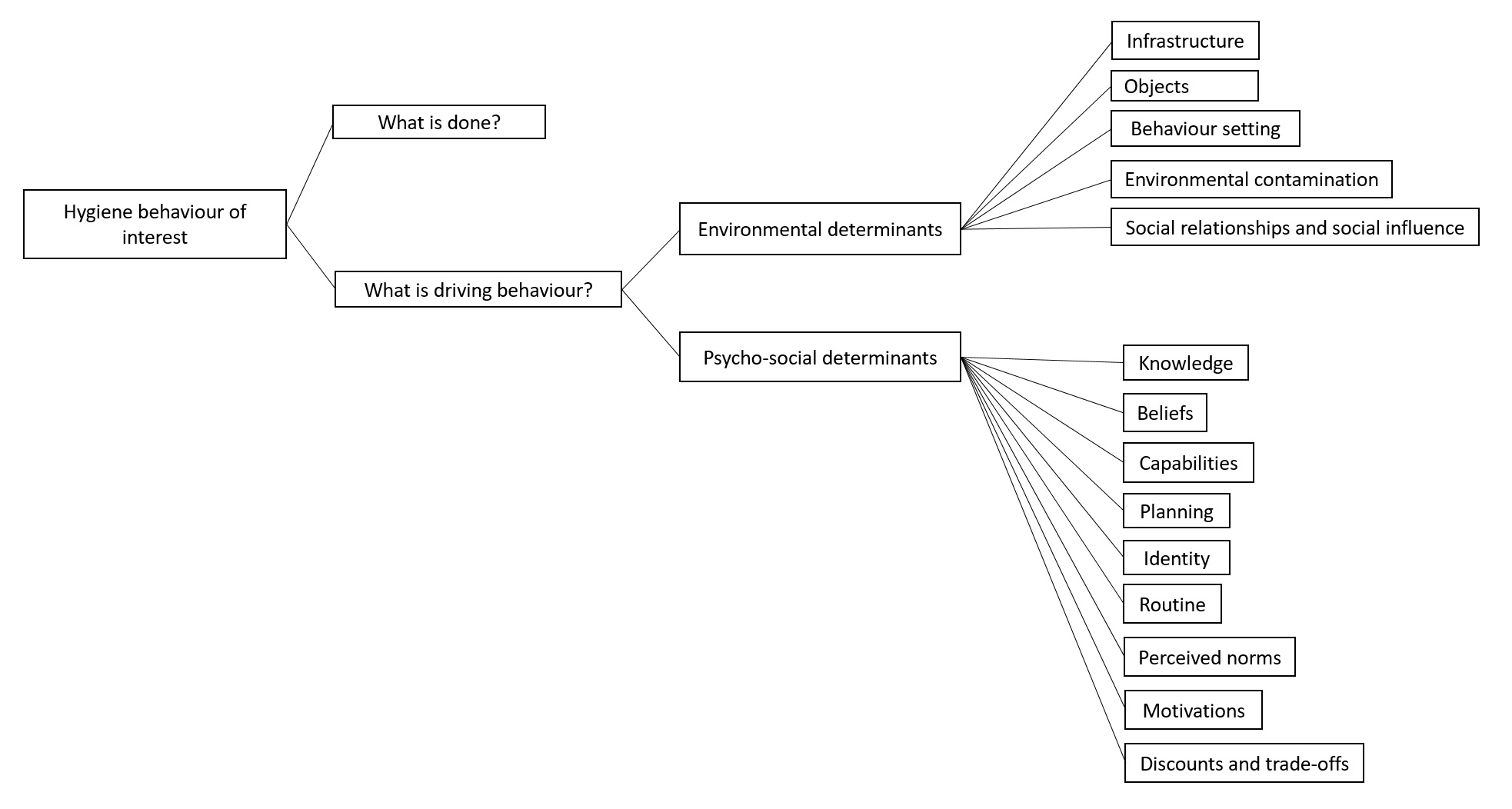

Supplement: S1 Fig — (TIF) [file pntd.0007784.s001.tif]
